# Assessment of *RAS* Dependency for *BRAF* Alterations Using Cancer Genomic Databases

**DOI:** 10.1001/jamanetworkopen.2020.35479

**Published:** 2021-01-28

**Authors:** Yiqing Zhao, Hanzhong Yu, Cris M. Ida, Kevin C. Halling, Benjamin R. Kipp, Katherine Geiersbach, Kandelaria M. Rumilla, Sounak Gupta, Ming-Tseh Lin, Gang Zheng

**Affiliations:** 1 Department of Health Sciences Research, Mayo Clinic, Rochester, Minnesota; 2 Department of Pathology and Laboratory Medicine, Mayo Clinic, Rochester, Minnesota; 3 Department of Pathology, Johns Hopkins University School of Medicine, Baltimore, Maryland

## Abstract

**Importance:**

Understanding *RAS* dependency and mechanisms of *RAS* activation in non-V600 *BRAF* variant cancers has important clinical implications. This is the first study to date to systematically assess *RAS* dependency of *BRAF* alterations with real-world cancer genomic databases.

**Objective:**

To evaluate *RAS* dependency of individual *BRAF* alterations through alteration coexistence analysis using cancer genomic databases.

**Design and Setting:**

A cross-sectional data analysis of 119 538 nonredundant cancer samples using cancer genomics databases including GENIE (Genomics Evidence Neoplasia Information Exchange) and databases in cBioPortal including TCGA (The Cancer Genome Atlas) (accessed March 24, 2020), in addition to 2745 cancer samples from Mayo Clinic Genomics Laboratory (January 1, 2015, to July 1, 2020). Frequencies and odds ratios of coexisting alterations of *RAS* (*KRAS*, *NRAS* and *HRAS*) and *RAS* regulatory genes (*NF1*, *PTPN11* and *CBL*) were calculated for individual *BRAF* alterations, and compared according to the current *BRAF* alteration classification; cancer type specificity of coexisting alterations of *RAS* or *RAS* regulatory genes was also evaluated.

**Main Outcomes and Measures:**

Primary outcome measurement is enrichment of *RAS* (*KRAS*, *NRAS* and *HRAS*) alterations in *BRAF* variant cancers. Secondary outcome measurement is enrichment of *RAS* regulatory gene (*NF1*, *PTPN11*, and *CBL*) in *BRAF* variant cancers.

**Results:**

A total of 2745 cancer samples from 2708 patients (female/male ratio: 1.0) tested by Mayo Clinic Genomics Laboratory and 119 538 patients (female/male ratio: 1.1) from GENIE and cBioPortal database were included in the study. In 119 538 nonredundant cancer samples, class 1 *BRAF* alterations and *BRAF* fusions were found to be mutually exclusive to alterations of *RAS* or *RAS* regulatory genes (odds ratio range 0.03-0.13 and 0.03-0.73 respectively), confirming their *RAS* independency. Both class 2 and class 3 *BRAF* alterations show variable and overlapping levels of enriched *RAS* alterations (odds ratio range: 0.03-5.9 and 0.63-2.52 respectively), suggesting heterogeneity in *RAS* dependency and a need to revisit *BRAF* alteration classification. For *RAS*-dependent *BRAF* alterations, the coexisting alterations also involve *RAS* regulatory genes by enrichment analysis (for example, S467L shows an odds ratio of 8.26 for *NF1*, 9.87 for *PTPN11*, and 15.23 for *CBL*) and occur in a variety of cancer types with some coalterations showing cancer type specificity (for example, *HRAS* variations account for 46.7% of all coexisting RAS alterations in BRAF variant bladder cancers, but 0% in non–small cell lung cancers). Variant-level assessment shows that *BRAF* alterations involving the same codon may differ in RAS dependency. In addition, *RAS* dependency of previously unclassified *BRAF* alterations could be assessed.

**Conclusions and Relevance:**

Current *BRAF* alteration classification based on in vitro assays does not accurately predict RAS dependency in vivo for non-V600 *BRAF* alterations. *RAS*-dependent *BRAF* variant cancers with different mechanisms of *RAS* activation suggest the need for different treatment strategies.

## Key Points

**Question:**

What is the in vivo *RAS* dependency of *BRAF* alterations in cancer samples?

**Findings:**

In this cross-sectional study of genomic data analysis with more than 11 9000 cancer samples, it was found that current *BRAF* alteration classification does not accurately represent in vivo *RAS* dependency for non-V600 alterations.

**Meaning:**

Results of this study suggest a need to revisit the classification of *BRAF* alterations, which has important clinical implications because personalized treatment strategies could be developed for *RAS*-dependent *BRAF* variant cancers based on different mechanisms of *RAS* activation.

## Teaser

This cross-sectional study assesses existing *BRAF* alteration classifications and the spectrum of coexisting alterations of *RAS* pathway genes and cancer types in which they occur using nonredundant cancer samples from cancer genomics databases.

## Introduction

*BRAF* alterations have been classified into class 1, 2, and 3 based on dimer formation, enzyme activity, and *RAS* dependence from in vitro experiments.^1,2^ Different classes of *BRAF* alterations differ in mechanism of action and require different treatment strategies.^3,4,5,6^ Class 3 alterations are different from class 1 (V600) and class 2 (non-V600 activating alterations) in that they are *RAS*-dependent with impaired kinase activity.^1,2^ Class 2 *BRAF* alterations were defined as *RAS* independent, and as activating *BRAF* alterations they are expected to be mutually exclusive to *RAS* alterations similar to class 1 *BRAF* alterations. However, some class 2 *BRAF* alterations were reported to coexist with *RAS* alterations.^7,8^ In contrast, class 3 *BRAF* variants, defined as *RAS* dependent, can be found without coexisting *RAS* alterations.^7,8^ Variation coexistence analysis using cancer genomics database provides evidence for functional interactions between activated *RAS* and *BRAF* alterations, and thus can support in vivo *RAS* dependency or independency. However, such analysis requires a large cancer genomics database, and most published clinical studies are limited in size to systematically assess *RAS* dependency with real-world clinical data.

Herein with 119 538 nonredundant cancer samples in GENIE (Genomics Evidence Neoplasia Information Exchange)^5^ and cancer alteration databases in cBioPortal including TCGA (The Cancer Genome Atlas) (accessed March 24, 2020), in addition to 2745 cancer samples sequenced at Mayo Clinic Genomics Laboratory (January 1, 2015, to July 1, 2020), we systematically assessed *RAS* dependence of *BRAF* alterations by examining their coexisting alterations of *RAS* and other *RAS* regulatory genes including *NF1*, *PTPN11*, and *CBL*. We aim to evaluate existing BRAF alteration classifications and understand the spectrum of coexisting alterations of *RAS* pathway genes and cancer types in which they occur. In addition, leveraging the size of the database, variant level assessment of *RAS* dependency can be performed for some previously unclassified *BRAF* alterations.

## Methods

Targeted next generation sequencing data from 2745 cancer samples (lung cancer, colorectal cancer, and melanoma) that have undergone clinical testing at Mayo Clinic Genomics Laboratory from January 1, 2015, to July 1, 2020, were collected with ACE (Advanced Cohort Explorer). This study was approved by the institutional review board at Mayo Clinic Rochester. A waiver was granted by the institutional review board at Mayo Clinic Rochester because the study is a retrospective data analysis with deidentified data. In addition, targeted next generation sequencing data from 79 720 samples from 75 191 patients in GENIE database (v.7.0, public release: January 2020, access date: March 24, 2020), and 176 nonduplicate studies available in cBioPortal (access date: March 24, 2020)^9,10^ including 46 697 samples from 44 347 patients were also collected. Both GENIE and cBioPortal databases are publicly available to the entire scientific community. We removed duplicated patients and only kept their earliest record, resulting in a total number of 119 538 non-Mayo samples. We removed duplicate records and kept only the earliest record, resulting in a total number of 119 538 non-Mayo samples.

### Statistical Analysis

To analyze the coexistence pattern of genes of interest (*KRAS*, *NRAS*, *HRAS*, *NF1*, *PTPN11*, *CBL*), distributions of samples based on *BRAF* alteration status (*BRAF altered* and *BRAF unaltered*) and alteration status of genes of interest were summarized in 2 × 2 contingency tables. The SNVs and indels, fusions (for *BRAF*), and copy number alterations (*RAS* amplification and *NF1* loss) were included in the analysis. Variants with less than 2 Catalogue of Somatic Mutations in Cancer counts were excluded as passenger alterations. Odds ratios (ORs) and frequencies of coalterations were calculated for individual *BRAF* alterations. *BRAF* alteration classification was based on previous studies.^1,2^ The χ^2^ test was used with the 2 × 2 contingency tables to test whether alterations coexist or are mutually exclusive to each other. The 95% CIs of ORs were calculated as exp(ln(OR)±1.96*SE(ln(OR))). Standard error SE(ln(OR)) was calculated as [(1/*A*) + (1/*B*) + (1/*C*) + (1/*D*)]^1/2^, where A, B, C, D are 4 numbers in the 2 × 2 contingency table. For contingency table with a value of 0, we applied Haldane-Anscombe correction by adding 0.05 to each cell and then calculated OR and its CI. *P* value was calculated through χ^2^ test for each contingency table. A *P* < .05 was considered statistically significant. This study followed the Strengthening the Reporting of Observational Studies in Epidemiology (STROBE) reporting guideline for cross-sectional studies as applicable.

## Results

**Figure 1.**
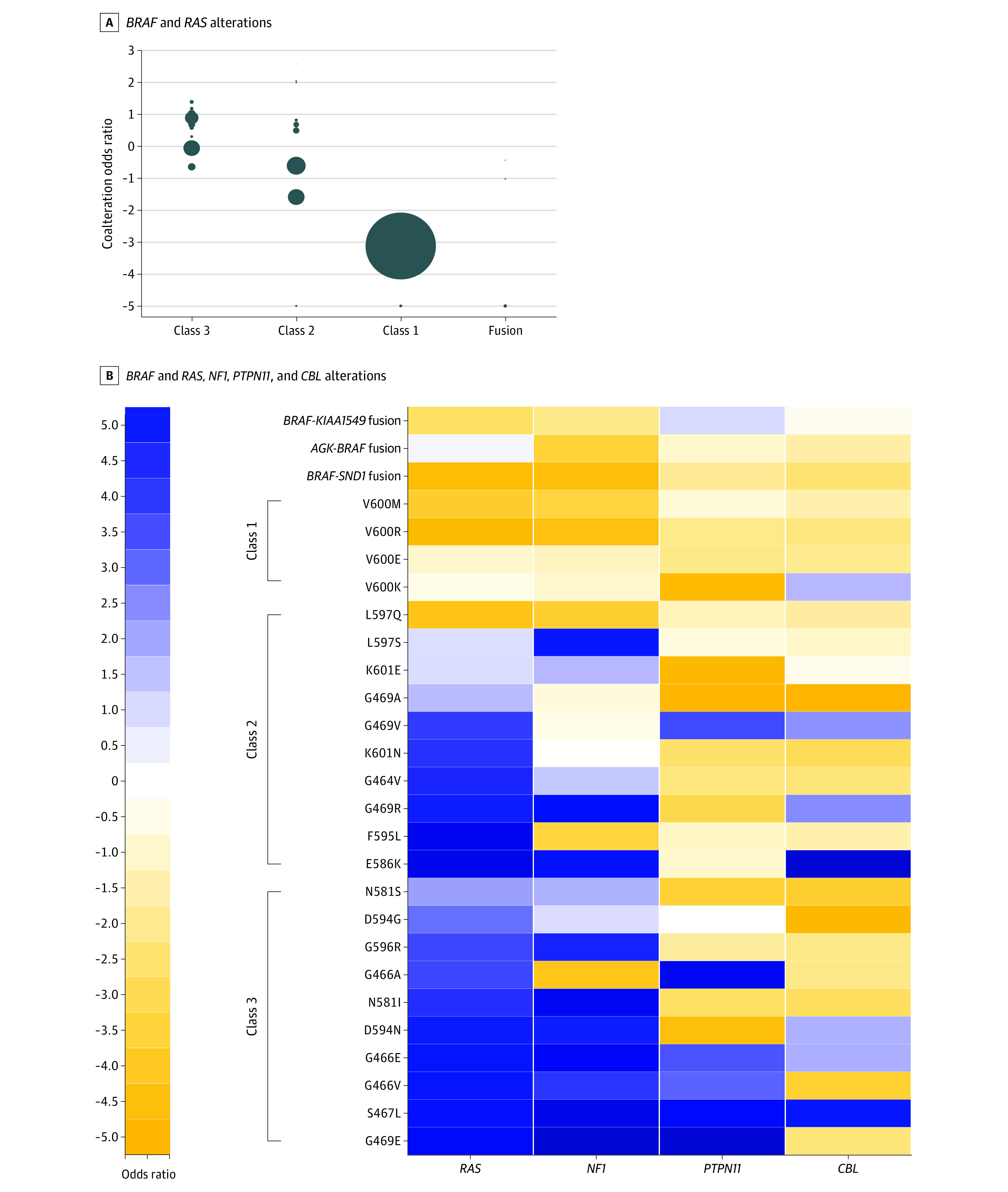
Summary of Coexisting Alterations of *RAS* and *RAS* Regulatory Genes for Individual *BRAF* Alterations A, Coalteration associations between *BRAF* alterations and *RAS* (*KRAS*, *NRAS*, and *HRAS*) alterations. Individual dots represent different alterations in the class. Size of dots represents numbers of patients with this alteration. B, Coalteration associations between *BRAF* alterations and *RAS*, *NF1*, *PTPN11*, and *CBL* alterations. Colors represent log2 odds ratio, and positive values were normalized to a −5 to 0 range, and negative values were normalized to a 0 to 5 range.

A total of 2745 cancer samples from 2708 patients (female/male ratio: 1.0) tested by Mayo Clinic Genomics Laboratory and 119 538 patients (female/male ratio: 1.1) from GENIE and cBioPortal database were included in the study. Targeted gene alteration data from 2708 patients tested by Mayo Clinic Genomics Laboratory showed that both class 3 and class 2 *BRAF* alterations have higher frequencies of coexisting *RAS* alterations than class 1 (10 of 38 [26.3%] for class 3, 4 of 22 [18.1%] for class 2, and 1 of 304 [0.3%] for class 1; *P* < .001 by χ^2^ for both class 3 vs class 1 and class 2 vs class 1). The Table lists the *BRAF* alterations that coexist with *RAS* alterations among the Mayo Clinic tested patient cohort. Shown by large genomic database analysis using GENIE and cancer alteration databases in cBioPortal, 5767 cases harbored *BRAF* alterations, 3761 of which belong to class 1, 529 class 2, 651 class 3, and 282 *BRAF* fusions, with the remaining unclassifiable. Among them, 138 (3.6%) cases with class 1 *BRAF* alterations, 107 (20.2%) class 2, 212 (32.6%) class 3, and 8 (2.8%) cases with *BRAF* fusions harbor alterations in *RAS* or *RAS* regulatory genes. As shown in Figure 1, both *BRAF* class 1 alterations and fusions are mutually exclusive to coexisting *RAS* alterations (*P* < .001 by χ^2^ for both; odds ratios range from 0.003-0.13 and 003-0.73, respectively) (eTable in the Supplement),^2,11^ but class 2 and class 3 alterations show heterogeneous and overlapping levels of ORs for coexisting *RAS* alterations (odds ratio range: 0.03-5.9 and 0.63-2.52 respectively) (Figure 1A). Some class 2 alterations show high ORs of *RAS* coalteration supporting their RAS dependence (eg, p.E586K [OR, 3.8; 95% CI, 1.6-9.2] and p.F595L [OR, 3.5; 95% CI, 1.5-8.4]). Some class 3 *BRAF* alterations (such as N581S) show lower OR (0.63) of coexisting *RAS* alterations than many class 2 alterations (Figure 1A-B and the eTable in the Supplement).

**Table.** *BRAF*-*RAS* Coalteration Cases From Mayo Clinic Cohort

Case	Diagnosis	*BRAF* alteration	*RAS* alteration	*BRAF* alteration class
1	Metastatic colorectal adenocarcinoma	p.V600E	*KRAS* p.G12V	1
2	Lung adenocarcinoma	p.L597Q	*NRAS* p.Q61R	2
3	Histiocytic sarcoma	p.K601N	*NRAS* p.Q61K	2
4	Melanoma	p.Leu597Ser	*NRAS* p.G13R	2
5	Colorectal adenocarcinoma	p.Gly469Val	*KRAS* p.G12S	2
6	Invasive high-grade urothelial carcinoma	p.D594G	*KRAS* p.G12V	3
7	Lung adenocarcinoma	p.N581I	*KRAS* p.G12S	3
8	Metastatic lung adenocarcinoma	p.D594H	*KRAS* p.G12V	3
9	Metastatic melanoma	p.G466E	*NRAS* p.A146V	3
10	Metastatic melanoma	p.G466V	*NRAS* p.A146V	3
11	Soft tissue, melanoma	p.S467L	*NRAS* p.Q61R	3
12	Metastatic melanoma	p.S467L	*NRAS* p.Q61R	3
13	Metastatic melanoma	p.G466R	*NRAS* p.Q61H	3
14	Metastatic melanoma	p.G596C	*NRAS* p.Q61L	3
15	Melanoma	p.D594E	*NRAS* p.Q61H	3
16	Lung adenocarcinoma	p.G596V	*KRAS* p.G13C	NA

Abbreviation: NA, not applicable.

**Figure 2.**
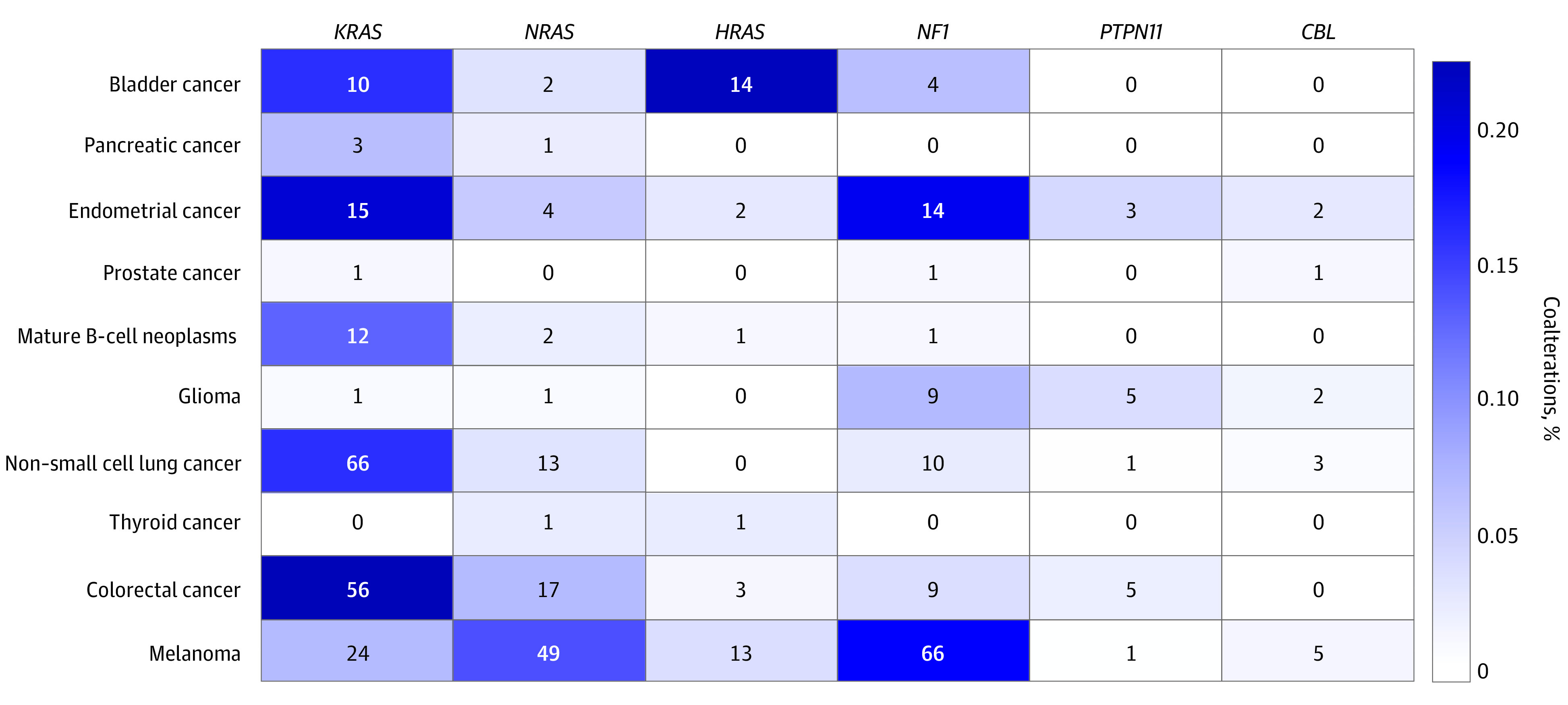
Cancer Type Distributions of Coexisting Mutations of *RAS* or *RAS* Regulatory Genes for *BRAF* Mutations Numbers represent sample counts. Colors reflect percentage of coalterations among all *BRAF* alterations within the cancer type.

For *BRAF* alterations showing *RAS* dependency, coalterations of *NF1*, *PTPN11* and *CBL* also exist (Figure 1B), supporting a variety of mechanisms of *RAS* activation in these tumors. With the variant level assessment, our study suggests alterations involving the same codon may have different *RAS* dependency. For example, p.G469E shows much higher frequency of coalterations of *RAS* or *RAS* regulatory genes than p.G469A (20 of 34 [58.8%] vs 21 of 162 [13.0%]; *P* < .001 by χ^2^) (Figure 1B). In addition, *RAS* dependency manifested as enriched alterations of *RAS* or *RAS* regulatory genes is a common phenomenon in various types of tumors (Figure 2), not limited to melanoma as previously suggested.^2^ Relative cancer type-specific coalterations exist. While *KRAS* is the overall dominant gene that is coaltered, *HRAS* contributed to most coalteration cases in bladder cancer, and *NF1* coalteration preferably occurs in melanoma and endometrial cancer. In addition, our study assessed *RAS* dependency of previously unclassified alterations. For example, p.E501K (OR, 4.3; 95% CI, 1.9-10.0), p.S363F (OR, 3.4; 95% CI, 1.0-12.0), p.R354Q (OR, 3.2; 95% CI, 1.1-9.9), and p.E26D (OR, 2.5; 95% CI, 1.2-5.1) show enriched coexisting *RAS* or *RAS* regulatory genes (eTable in the Supplement), suggesting that they are *RAS* dependent.

## Discussion

Findings of this cross-sectional study suggest *RAS* independency in class 1 *BRAF* (V600) alterations and that enriched coexisting *RAS* alterations for some class 3 *BRAF* alterations support *RAS* dependency. In addition, our study presents an extended spectrum of coexisting alterations of *RAS* regulatory genes including *NF1*, *PTPN11*, and *CBL*, indicating different mechanisms of activating *RAS* for *RAS*-dependent *BRAF* variant tumors. This study also shows heterogeneity in *RAS* dependency in class 2 and 3 alterations, which cannot be explained by current *BRAF* alteration classification. Classification of *BRAF* alterations was mostly achieved by in vitro assays and *RAS* dependency established with mouse embryonal fibroblasts established in literature.^12,13^ This study suggests that they may not accurately predict in vivo function. Thus, revisiting the classification of *BRAF* alterations is warranted. In addition, our study shows that coexisting alterations of *RAS* or *RAS* regulatory gene are common phenomena in various types of tumors, more broadly than previous recognized.^2^ Moreover, the variant level assessment can be used to evaluate previously unclassified alterations and is important in deciphering its clinical significance and enabling precision medicine.

Our study has notable clinical implications. First, class 2 but not class 3 *BRAF* alterations may respond to MEK inhibitors like trametinib.^3,4,5,6^ Our study showed that some class 2 *BRAF* alterations are actually *RAS* dependent and may behave similarly to class 3 alterations and not respond to MEK inhibitors. Second, *RAS* dependency suggests targeting *RAS* or signaling upstream may be effective, and the presence of coexisting alterations may affect treatment selection. For those without coexisting *RAS* or *NF1* alterations, upstream signaling including EGFR,^2^ tyrosine kinase receptor,^14^ and SHP2^3^ may be targeted. *PTPN11* and *CBL* are potentially targetable if their alterations are present. If *RAS* or *NF1* coalterations are present, however, targeting downstream signaling including *RAF* (such as sorafnib)^15^ may be more effective. In addition, our study also suggests that when a *BRAF* alteration with *RAS* dependency is identified, reflex testing should be performed to assess alteration status of *RAS* and *RAS* regulatory genes, which may lead to more precise assessment of therapeutic vulnerabilities and strategies. These study findings suggest that large cancer genomics data analysis provides evidence-based gene alteration interpretation to assist in clinical decision-making in the context of precision medicine.

### Limitations

This study has several limitations. First, clonal architecture of the cancer samples could not be fully assessed with the available single-time point genomic data; in theory, the coexisting alterations could be present in different tumor subclones, though a previous study showed that concomitant *BRAF* and *RAS* alterations were mostly present in the same tumor cell populations.^16^ Second, some rare non-V600 *BRAF* alterations are underrepresented in the genomic databases; thus their *RAS* dependency cannot be reliably assessed with the approach in the study. In addition, for analysis of individual *BRAF* alterations, multiple-testing-inflated false discoveries could not be fully excluded. Third, this is a cross-sectional data mining study, and future laboratory and clinical research are needed to confirm the findings in the study.

## Conclusions

These findings suggest that there are different mechanisms of *RAS* activation for *RAS*-dependent *BRAF* alterations in a wide variety of cancer types, including alterations of *RAS* genes and *RAS* regulatory genes. In addition, current *BRAF* alteration classification based on in vitro assays does not accurately predict *RAS* dependency in vivo for non-V600 *BRAF* alterations.

## Notes

Supplement. **eTable.** Frequencies and Odds Ratios of Coexisting Mutations of KRAS, NRAS, HRAS, NF1, PTPN11 and CBL for all BRAF Mutations With ≥5 Count
