# Supplementary material for: Assessment of RAS Dependency for BRAF Alterations Using Cancer Genomic Databases
Source: JAMA Netw Open. 2021 Jan 28;4(1):e2035479. doi: 10.1001/jamanetworkopen.2020.35479 (PMC7844594; doi:10.1001/jamanetworkopen.2020.35479)
Supplement: Supplement. — eTable. Frequencies and Odds Ratios of Coexisting Mutations of KRAS, NRAS, HRAS, NF1, PTPN11 and CBL for all BRAF Mutations With ≥5 Count [file jamanetwopen-e2035479-s001.pdf]

Supplemental Online Content

Zhao Y, Yu H, Ida CM, et al. Assessment of *RAS* dependency for *BRAF* alterations using cancer genomic databases. *JAMA Netw Open*. 2021;4(1):e2035479. doi:10.1001/jamanetworkopen.2020.35479

eTable. Frequencies and Odds Ratios of Coexisting Mutations of KRAS, NRAS, HRAS, NF1, PTPN11 and CBL for all BRAF Mutations With ≥5 Count

This supplemental material has been provided by the authors to give readers additional information about their work.

© 2021 Zhao Y et al. *JAMA Network Open*.

eTable 1 Frequencies and odds ratios of coexisting mutations of KRAS, NRAS, HRAS, NF1, PTPN11 and CBL for all BRAF mutations with ≥5 count

	Count	KRAS						NRAS					
		Altered	OR	SE(ln(OR))	ci_upper	ci_lower	P_value	Altered	OR	SE(ln(OR))	ci_upper	ci_lower	P_value
V600E	3607	30	0.06	0.18	0.08	0.04	0.000	40	0.44	0.16	0.61	0.32	0.000
G469A	162	15	0.66	0.27	1.12	0.39	0.150	3	0.74	0.58	2.33	0.24	0.794
K601E	143	5	0.25	0.46	0.61	0.10	0.001	3	0.84	0.58	2.64	0.27	0.976
D594G	142	15	0.75	0.27	1.28	0.44	0.352	4	1.13	0.51	3.05	0.42	0.976
V600K	134	1	0.05	1.00	0.38	0.01	0.000	2	0.60	0.71	2.42	0.15	0.651
D594N	116	20	1.23	0.24	1.97	0.76	0.471	11	3.80	0.32	7.06	2.05	0.000
N581S	65	2	0.22	0.72	0.90	0.05	0.033	5	3.08	0.46	7.66	1.24	0.030
G466V	64	18	2.00	0.27	3.38	1.19	0.013	3	1.88	0.59	5.98	0.59	0.491
G466E	59	11	1.33	0.33	2.53	0.70	0.493	4	2.72	0.52	7.48	0.99	0.108
G469V	55	7	0.91	0.40	1.99	0.41	0.959	6	4.45	0.43	10.36	1.91	0.001
G469R	48	9	1.34	0.36	2.72	0.66	0.550	2	1.67	0.72	6.87	0.41	0.795
N581I	36	6	1.19	0.44	2.82	0.50	0.877	1	1.11	1.01	8.12	0.15	0.669
G469E	34	10	2.10	0.36	4.24	1.03	0.061	5	5.89	0.48	15.08	2.30	0.000
K601N	34	6	1.26	0.44	2.99	0.53	0.782	2	2.36	0.73	9.82	0.57	0.500
P403Lfs*8	31	11	2.53	0.35	5.03	1.27	0.012	1	1.29	1.02	9.47	0.18	0.749
S467L	29	1	0.25	1.02	1.80	0.03	0.223	10	13.82	0.37	28.38	6.73	0.000
G464V	27	7	1.85	0.42	4.24	0.80	0.227	1	1.48	1.02	10.93	0.20	0.824
L597R	27	1	0.26	1.02	1.94	0.04	0.263	2	2.97	0.73	12.49	0.71	0.339
BRAF-KIAA1549 fusion	55	0	0.01	4.48	88.36	0.00	0.067	0	0.08	4.48	497.06	0.00	1.000
T599dup	26	0	0.01	4.48	88.36	0.00	0.067	0	0.08	4.48	497.06	0.00	1.000
N486_P490del	26	0	0.01	4.48	88.36	0.00	0.067	0	0.08	4.48	497.06	0.00	1.000
V600R	25	0	0.01	4.48	91.92	0.00	0.065	0	0.08	4.48	517.07	0.00	1.000
G466A	24	2	0.59	0.74	2.51	0.14	0.676	3	5.01	0.61	16.64	1.51	0.021
E26D	23	8	2.48	0.41	5.54	1.11	0.044	0	0.09	4.48	562.37	0.00	1.000

© 2021 Zhao Y et al. *JAMA Network Open*.

	Count	KRAS						NRAS					
		Altered	OR	SE(ln(OR))	ci_upper	ci_lower	P_value	Altered	OR	SE(ln(OR))	ci_upper	ci_lower	P_value
G466R	23	0	0.02	4.48	99.97	0.00	0.104	5	8.71	0.49	22.93	3.31	0.000
A404Cfs*9	23	1	0.31	1.02	2.29	0.04	0.366	2	3.48	0.74	14.79	0.82	0.247
BRAF-SND1 fusion	22	0	0.02	4.48	104.55	0.00	0.101	0	0.09	4.48	588.12	0.00	1.000
G596R	20	1	0.36	1.02	2.65	0.05	0.471	1	2.00	1.02	14.93	0.27	0.987
V600_K601delinsE	20	0	0.02	4.48	115.09	0.00	0.162	1	2.00	1.02	14.93	0.27	0.987
L597Q	17	0	0.02	4.48	135.60	0.00	0.255	0	0.12	4.48	762.81	0.00	1.000
F595L	13	5	2.74	0.53	7.69	0.98	0.101	4	12.33	0.57	37.84	4.02	0.000
A762V	13	4	2.19	0.57	6.72	0.71	0.299	0	0.15	4.48	1000.55	0.00	1.000
E501K	12	6	3.56	0.50	9.49	1.34	0.018	1	3.34	1.04	25.69	0.43	0.741
E586K	12	3	1.78	0.65	6.31	0.50	0.608	5	16.70	0.53	47.42	5.88	0.000
D594E	12	2	1.19	0.76	5.30	0.27	0.856	1	3.34	1.04	25.69	0.43	0.741
AGK-BRAF fusion	12	0	0.03	4.48	192.89	0.00	0.383	1	3.34	1.04	25.69	0.43	0.741
R389C	11	2	1.30	0.77	5.84	0.29	0.932	1	3.64	1.04	28.22	0.47	0.697
V600M	11	0	0.03	4.48	210.69	0.00	0.380	0	0.18	4.48	1185.23	0.00	1.000
K483E	10	1	0.71	1.05	5.56	0.09	0.894	4	16.03	0.59	51.13	5.02	0.000
T241M	10	3	2.14	0.66	7.77	0.59	0.448	0	0.20	4.48	1305.73	0.00	1.000
E695Q	10	2	1.42	0.77	6.50	0.31	0.984	0	0.20	4.48	1305.73	0.00	1.000
L597S	10	0	0.04	4.48	232.11	0.00	0.623	0	0.20	4.48	1305.73	0.00	1.000
G469S	10	0	0.04	4.48	232.11	0.00	0.623	0	0.20	4.48	1305.73	0.00	1.000
R271H	9	3	2.37	0.67	8.77	0.64	0.369	0	0.22	4.48	1453.50	0.00	1.000
D594Y	9	4	3.17	0.60	10.28	0.97	0.109	1	4.45	1.05	35.15	0.56	0.599
L485W	9	0	0.04	4.48	258.38	0.00	0.613	0	0.22	4.48	1453.50	0.00	1.000
R354Q	8	0	0.04	4.49	291.35	0.00	0.607	0	0.25	4.49	1638.97	0.00	1.000
L584F	8	2	1.78	0.79	8.39	0.38	0.796	0	0.25	4.49	1638.97	0.00	1.000
D594H	8	2	1.78	0.79	8.39	0.38	0.796	0	0.25	4.49	1638.97	0.00	1.000
BRAF-MKRN1 fusion	8	1	0.89	1.06	7.12	0.11	0.691	0	0.25	4.49	1638.97	0.00	1.000
D594A	7	2	2.04	0.80	9.80	0.42	0.691	0	0.28	4.49	1878.66	0.00	1.000

© 2021 Zhao Y et al. *JAMA Network Open*.

	Count	KRAS						NRAS					
		**Altered**	**OR**	**SE(ln(OR))**	**ci_upper**	**ci_lower**	**P_value**	**Altered**	**OR**	**SE(ln(OR))**	**ci_upper**	**ci_lower**	**P_value**
R671Q	7	0	0.05	4.49	333.96	0.00	1.000	0	0.28	4.49	1878.66	0.00	1.000
S363F	6	0	0.06	4.49	391.15	0.00	1.000	0	0.33	4.49	2200.42	0.00	1.000
G464R	6	2	2.37	0.82	11.77	0.48	0.579	0	0.33	4.49	2200.42	0.00	1.000
L597V	5	3	4.27	0.73	17.89	1.02	0.103	2	16.03	0.84	82.65	3.11	0.001
P708S	5	1	1.42	1.10	12.20	0.17	0.767	0	0.40	4.49	2655.03	0.00	1.000
R509*	5	2	2.85	0.84	14.69	0.55	0.463	0	0.40	4.49	2655.03	0.00	1.000
D594V	5	3	4.27	0.73	17.89	1.02	0.103	0	0.40	4.49	2655.03	0.00	1.000
N581T	5	0	0.07	4.49	471.97	0.00	1.000	0	0.40	4.49	2655.03	0.00	1.000
F468S	5	0	0.07	4.49	471.97	0.00	1.000	0	0.49	4.50	3346.06	0.00	1.000
G596C	5	1	1.42	1.10	12.20	0.17	0.767	0	0.40	4.49	2655.03	0.00	1.000
BRAF-CDK5RAP2 fusion	5	0	0.07	4.49	471.97	0.00	1.000	0	0.40	4.49	2655.03	0.00	1.000
AGAP3-BRAF fusion	5	0	0.07	4.49	471.97	0.00	1.000	0	0.40	4.49	2655.03	0.00	1.000

© 2021 Zhao Y et al. *JAMA Network Open*.

	Count	HRAS						NF1					
		Altered	OR	SE(ln(OR))	ci_upper	ci_lower	P_value	Altered	OR	SE(ln(OR))	ci_upper	ci_lower	P_value
V600E	3607	4	0.19	0.50	0.51	0.07	0.000	49	0.67	0.15	0.89	0.50	0.00
G469A	162	1	1.05	1.00	7.54	0.15	0.614	3	0.87	0.58	2.74	0.28	1.00
K601E	143	0	0.06	4.47	382.96	0.00	1.000	6	2.10	0.42	4.79	0.92	0.07
D594G	142	4	4.81	0.51	13.02	1.77	0.011	5	1.74	0.46	4.26	0.71	0.22
V600K	134	0	0.06	4.47	408.71	0.00	1.000	2	0.76	0.71	3.09	0.19	1.00
D594N	116	6	8.83	0.42	20.13	3.87	0.000	12	4.81	0.31	8.76	2.64	0.00
N581S	65	0	0.13	4.47	843.70	0.00	1.000	3	2.14	0.59	6.85	0.67	0.17
G466V	64	1	2.67	1.01	19.25	0.37	0.316	5	3.85	0.47	9.65	1.53	0.01
G466E	59	1	2.89	1.01	20.91	0.40	0.296	9	7.07	0.36	14.38	3.47	0.00
G469V	55	2	6.32	0.72	25.98	1.54	0.042	1	0.96	1.01	6.96	0.13	1.00
G469R	48	2	7.11	0.72	29.32	1.72	0.035	6	6.04	0.44	14.28	2.55	0.00
N581I	36	2	9.48	0.73	39.46	2.28	0.021	5	7.55	0.49	19.67	2.90	0.00
G469E	34	0	0.25	4.48	1616.78	0.00	1.000	5	10.33	0.50	27.69	3.85	0.00
K601N	34	1	5.02	1.02	36.72	0.69	0.185	1	1.23	1.02	8.98	0.17	0.56
P403Lfs*8	31	0	0.27	4.48	1774.09	0.00	1.000	3	3.80	0.60	12.43	1.16	0.05
S467L	29	0	0.29	4.48	1897.15	0.00	1.000	4	8.26	0.55	24.31	2.81	0.00
G464V	27	0	0.32	4.48	2038.56	0.00	1.000	1	1.87	1.02	13.90	0.25	0.43
L597R	27	2	12.64	0.73	53.27	3.00	0.012	2	3.57	0.74	15.18	0.84	0.12
BRAF-KIAA1549 fusion	55	0	0.33	4.48	2117.47	0.00	1.000	1	1.51	1.02	11.13	0.20	0.49
T599dup	26	0	0.33	4.48	2117.47	0.00	1.000	0	0.08	4.48	527.79	0.00	1.00
N486_P490del	26	0	0.34	4.48	2202.74	0.00	1.000	0	0.08	4.48	486.93	0.00	1.00
V600R	25	0	0.34	4.48	2202.74	0.00	1.000	0	0.10	4.48	634.23	0.00	1.00
G466A	24	0	0.35	4.48	2295.16	0.00	1.000	0	0.11	4.48	705.36	0.00	1.00
E26D	23	1	7.42	1.02	55.03	1.00	0.131	2	3.41	0.74	14.49	0.80	0.13
G466R	23	0	0.37	4.48	2395.68	0.00	1.000	3	6.54	0.62	22.22	1.93	0.01
A404Cfs*9	23	0	0.37	4.48	2395.68	0.00	1.000	2	3.41	0.74	14.49	0.80	0.13
BRAF-SND1	22	0	0.39	4.48	2505.41	0.00	1.000	0	0.09	4.48	576.14	0.00	1.00

© 2021 Zhao Y et al. *JAMA Network Open*.

	Count	HRAS						NF1					
		Altered	OR	SE(ln(OR))	ci_upper	ci_lower	P_value	Altered	OR	SE(ln(OR))	ci_upper	ci_lower	P_value
G596R	20	1	8.53	1.03	63.68	1.14	0.115	2	4.36	0.75	18.81	1.01	0.09
V600_K601delins E	20	0	0.43	4.48	2758.05	0.00	1.000	0	0.13	4.48	848.01	0.00	1.00
L597Q	17	0	0.50	4.48	3249.58	0.00	1.000	0	0.15	4.48	980.16	0.00	1.00
F595L	13	0	0.65	4.48	4262.34	0.00	1.000	0	0.18	4.48	1161.08	0.00	1.00
A762V	13	1	13.13	1.04	100.50	1.72	0.079	0	0.15	4.48	980.16	0.00	1.00
E501K	12	0	0.71	4.48	4622.49	0.00	1.000	3	9.81	0.65	34.80	2.77	0.01
E586K	12	0	0.71	4.48	4622.49	0.00	1.000	1	5.61	1.07	45.60	0.69	0.18
D594E	12	1	14.22	1.04	109.54	1.85	0.073	0	0.28	4.49	1840.38	0.00	1.00
AGK-BRAF	12	0	0.71	4.48	4622.49	0.00	1.000	0	0.16	4.48	1062.97	0.00	1.00
R389C	11	0	0.77	4.48	5049.10	0.00	1.000	2	7.14	0.77	32.22	1.58	0.04
V600M	11	0	0.77	4.48	5049.10	0.00	1.000	0	0.18	4.48	1161.08	0.00	1.00
K483E	10	0	0.85	4.48	5562.43	0.00	1.000	3	11.78	0.66	42.81	3.24	0.00
T241M	10	1	17.07	1.05	133.52	2.18	0.062	0	0.20	4.48	1279.12	0.00	1.00
E695Q	10	0	0.85	4.48	5562.43	0.00	1.000	0	0.20	4.48	1279.12	0.00	1.00
L597S	10	0	0.85	4.48	5562.43	0.00	1.000	1	4.91	1.06	39.25	0.61	0.20
G469S	10	0	0.85	4.48	5562.43	0.00	1.000	0	0.20	4.48	1279.12	0.00	1.00
R271H	9	1	18.96	1.05	149.89	2.40	0.057	1	4.36	1.05	34.44	0.55	0.22
D594Y	9	0	0.94	4.48	6191.93	0.00	1.000	0	0.28	4.49	1840.38	0.00	1.00
L485W	9	0	0.94	4.48	6191.93	0.00	1.000	0	0.22	4.48	1423.88	0.00	1.00
R354Q	8	2	42.67	0.79	201.30	9.04	0.001	5	24.53	0.57	75.04	8.02	0.00
L584F	8	0	1.06	4.49	6982.02	0.00	1.000	2	11.22	0.80	54.01	2.33	0.02
D594H	8	0	1.06	4.49	6982.02	0.00	1.000	0	0.39	4.49	2600.93	0.00	1.00

© 2021 Zhao Y et al. *JAMA Network Open*.

	**Count**	**HRAS**						**NF1**					
		**Altered**	**OR**	**SE(ln(OR))**	**ci_upper**	**ci_lower**	**P_value**	**Altered**	**OR**	**SE(ln(OR))**	**ci_upper**	**ci_lower**	**P_value**
BRAF-MKRN1 fusion	8	0	1.06	4.49	6982.02	0.00	1.000	0	0.24	4.49	1605.57	0.00	1.00
D594A	7	1	24.38	1.07	198.45	3.00	0.046	0	0.48	4.50	3277.87	0.00	1.00
R671Q	7	0	1.21	4.49	8003.12	0.00	1.000	2	11.22	0.80	54.01	2.33	0.02
S363F	6	2	56.89	0.82	282.38	11.46	0.001	2	13.08	0.82	64.86	2.64	0.02
G464R	6	0	1.41	4.49	9373.80	0.00	1.000	0	0.32	4.49	2155.57	0.00	1.00
L597V	5	0	1.69	4.49	11310.46	0.00	1.000	0	0.48	4.50	3277.87	0.00	1.00
P708S	5	0	1.69	4.49	11310.46	0.00	1.000	3	23.55	0.73	98.60	5.63	0.00
R509*	5	1	34.13	1.10	292.57	3.98	0.034	2	15.70	0.84	80.97	3.04	0.01
D594V	5	0	1.69	4.49	11310.46	0.00	1.000	0	0.39	4.49	2600.93	0.00	1.00
N581T	5	0	1.69	4.49	11310.46	0.00	1.000	1	9.81	1.12	87.83	1.10	0.12
F468S	5	0	2.11	4.50	14254.24	0.00	1.000	1	9.81	1.12	87.83	1.10	0.12
G596C	5	0	1.69	4.49	11310.46	0.00	1.000	0	0.96	4.53	6823.55	0.00	1.00
BRAF-CDK5RAP2 fusion	5	0	1.69	4.49	11310.46	0.00	1.000	0	0.39	4.49	2600.93	0.00	1.00
AGAP3-BRAF fusion	5	0	1.69	4.49	11310.46	0.00	1.000	0	0.39	4.49	2600.93	0.00	1.00

© 2021 Zhao Y et al. *JAMA Network Open*.

	Count	PTPN11						CBL					
		Altered	OR	SE(ln(OR))	ci_upper	ci_lower	P_value	Altered	OR	SE(ln(OR))	ci_upper	ci_lower	P_value
V600E	3607	7	0.53	0.38	1.12	0.25	0.091	9	0.87	0.34	1.69	0.45	0.874
G469A	162	0	0.09	4.47	558.87	0.00	1.000	0	0.11	4.47	682.00	0.00	1.000
K601E	143	0	0.10	4.47	610.83	0.00	1.000	1	2.47	1.01	17.74	0.34	0.335
D594G	142	1	1.93	1.00	13.84	0.27	0.406	0	0.12	4.47	771.87	0.00	1.000
V600K	134	0	0.10	4.47	657.88	0.00	1.000	2	5.54	0.72	22.54	1.36	0.053
D594N	116	0	0.12	4.47	763.77	0.00	1.000	2	5.77	0.72	23.50	1.42	0.049
N581S	65	0	0.21	4.47	1359.41	0.00	1.000	0	0.26	4.47	1663.08	0.00	1.000
G466V	64	1	4.30	1.01	31.07	0.59	0.210	0	0.27	4.47	1763.21	0.00	1.000
G466E	59	1	4.68	1.01	33.84	0.65	0.195	1	5.71	1.01	41.49	0.79	0.163
G469V	55	1	4.93	1.01	35.76	0.68	0.186	1	6.69	1.01	48.74	0.92	0.142
G469R	48	0	0.28	4.47	1823.85	0.00	1.000	1	6.85	1.01	49.99	0.94	0.139
N581I	36	0	0.37	4.48	2383.74	0.00	1.000	0	0.49	4.48	3156.92	0.00	1.000
G469E	34	2	16.15	0.73	67.53	3.86	0.008	0	0.68	4.48	4430.32	0.00	1.000
K601N	34	0	0.39	4.48	2524.66	0.00	1.000	0	0.46	4.48	2945.28	0.00	1.000
P403Lfs*8	31	0	0.43	4.48	2770.31	0.00	1.000	0	0.44	4.48	2849.76	0.00	1.000
S467L	29	1	9.87	1.02	72.80	1.34	0.100	1	15.23	1.03	114.38	2.03	0.067
G464V	27	0	0.49	4.48	3183.28	0.00	1.000	0	0.68	4.48	4430.32	0.00	1.000
L597R	27	0	0.51	4.48	3306.51	0.00	1.000	0	0.65	4.48	4217.66	0.00	1.000
BRAF-KIAA1549 fusion	55	0	0.51	4.48	3306.51	0.00	1.000	0	0.53	4.48	3401.34	0.00	1.000
T599dup	26	0	0.53	4.48	3439.66	0.00	1.000	0	0.59	4.48	3848.23	0.00	1.000
N486_P490del	26	0	0.53	4.48	3439.66	0.00	1.000	0	0.53	4.48	3401.34	0.00	1.000
V600R	25	0	0.55	4.48	3583.98	0.00	1.000	0	0.72	4.48	4665.55	0.00	1.000
G466A	24	1	11.10	1.02	82.27	1.50	0.089	0	0.80	4.48	5219.86	0.00	1.000
E26D	23	0	0.58	4.48	3740.94	0.00	1.000	0	0.59	4.48	3848.23	0.00	1.000
G466R	23	0	0.60	4.48	3912.28	0.00	1.000	0	0.72	4.48	4665.55	0.00	1.000
A404Cfs*9	23	0	0.58	4.48	3740.94	0.00	1.000	1	11.92	1.02	88.49	1.61	0.084

© 2021 Zhao Y et al. *JAMA Network Open*.

	Count	PTPN11						CBL					
		Altered	OR	SE(ln(OR))	ci_upper	ci_lower	P_value	Altered	OR	SE(ln(OR))	ci_upper	ci_lower	P_value
BRAF-SND1 fusion	22	0	0.60	4.48	3912.28	0.00	1.000	0	0.62	4.48	4024.49	0.00	1.000
G596R	20	0	0.66	4.48	4306.80	0.00	1.000	0	0.80	4.48	5219.86	0.00	1.000
V600_K601delinsE	20	0	0.66	4.48	4306.80	0.00	1.000	0	0.91	4.48	5923.62	0.00	1.000
L597Q	17	0	0.89	4.48	5758.47	0.00	1.000	0	1.05	4.48	6846.69	0.00	1.000
F595L	13	0	1.02	4.48	6655.80	0.00	1.000	0	1.24	4.48	8110.47	0.00	1.000
A762V	13	0	1.02	4.48	6655.80	0.00	1.000	0	1.05	4.48	6846.69	0.00	1.000
E501K	12	0	1.11	4.48	7218.18	0.00	1.000	0	1.14	4.48	7425.20	0.00	1.000
E586K	12	0	1.11	4.48	7218.18	0.00	1.000	1	34.26	1.06	274.67	4.27	0.032
D594E	12	0	1.21	4.48	7884.35	0.00	1.000	0	1.70	4.49	11215.36	0.00	1.000
AGK-BRAF fusion	12	0	1.11	4.48	7218.18	0.00	1.000	0	1.14	4.48	7425.20	0.00	1.000
R389C	11	3	72.68	0.65	261.46	20.20	0.000	1	24.92	1.05	193.53	3.21	0.043
V600M	11	0	1.21	4.48	7884.35	0.00	1.000	0	1.24	4.48	8110.47	0.00	1.000
K483E	10	0	1.33	4.48	8685.94	0.00	1.000	0	1.36	4.48	8935.05	0.00	1.000
T241M	10	0	1.33	4.48	8685.94	0.00	1.000	0	1.36	4.48	8935.05	0.00	1.000
E695Q	10	0	1.47	4.48	9668.92	0.00	1.000	0	1.51	4.48	9946.22	0.00	1.000
L597S	10	0	1.33	4.48	8685.94	0.00	1.000	0	1.70	4.49	11215.36	0.00	1.000
G469S	10	0	1.33	4.48	8685.94	0.00	1.000	0	1.36	4.48	8935.05	0.00	1.000
R271H	9	0	1.47	4.48	9668.92	0.00	1.000	0	1.51	4.48	9946.22	0.00	1.000
D594Y	9	0	1.47	4.48	9668.92	0.00	1.000	0	1.94	4.49	12855.57	0.00	1.000
L485W	9	0	1.47	4.48	9668.92	0.00	1.000	0	1.51	4.48	9946.22	0.00	1.000
R354Q	8	0	1.65	4.49	10902.67	0.00	1.000	1	34.26	1.06	274.67	4.27	0.032
L584F	8	0	1.65	4.49	10902.67	0.00	1.000	0	1.94	4.49	12855.57	0.00	1.000
D594H	8	0	1.65	4.49	10902.67	0.00	1.000	0	2.71	4.49	18168.22	0.00	1.000

© 2021 Zhao Y et al. *JAMA Network Open*.

	Count	PTPN11						CBL					
		**Altered**	**OR**	**SE(ln(OR))**	**ci_upper**	**ci_lower**	**P_value**	**Altered**	**OR**	**SE(ln(OR))**	**ci_upper**	**ci_lower**	**P_value**
BRAF-MKRN1 fusion	8	0	1.65	4.49	10902.67	0.00	1.000	0	1.70	4.49	11215.36	0.00	1.000
D594A	7	0	1.89	4.49	12497.15	0.00	1.000	0	3.38	4.50	22896.85	0.00	1.000
R671Q	7	0	1.89	4.49	12497.15	0.00	1.000	0	1.94	4.49	12855.57	0.00	1.000
S363F	6	0	2.20	4.49	14637.51	0.00	1.000	0	2.26	4.49	15057.32	0.00	1.000
G464R	6	0	2.20	4.49	14637.51	0.00	1.000	0	2.26	4.49	15057.32	0.00	1.000
L597V	5	0	2.64	4.49	17661.68	0.00	1.000	0	3.38	4.50	22896.85	0.00	1.000
P708S	5	1	53.30	1.10	457.19	6.21	0.022	0	2.71	4.49	18168.22	0.00	1.000
R509*	5	0	2.64	4.49	17661.68	0.00	1.000	0	2.71	4.49	18168.22	0.00	1.000
D594V	5	0	2.64	4.49	17661.68	0.00	1.000	0	2.71	4.49	18168.22	0.00	1.000
N581T	5	0	2.64	4.49	17661.68	0.00	1.000	1	68.53	1.12	614.65	7.64	0.018
F468S	5	0	2.64	4.49	17661.68	0.00	1.000	0	3.38	4.50	22896.85	0.00	1.000
G596C	5	0	3.29	4.50	22258.49	0.00	1.000	0	6.68	4.53	47664.30	0.00	1.000
BRAF-CDK5RAP2 fusion	5	0	2.64	4.49	17661.68	0.00	1.000	0	2.71	4.49	18168.22	0.00	1.000
AGAP3-BRAF fusion	5	0	2.64	4.49	17661.68	0.00	1.000	0	2.71	4.49	18168.22	0.00	1.000

© 2021 Zhao Y et al. *JAMA Network Open*.

	Count	Combined						
		Altered	OR	SE(ln(OR))	ci_upper	ci_lower	P_value	Q_value
V600E	3607	131	0.19	0.09	0.22	0.16	0.000	0.000
G469A	162	21	0.67	0.23	1.05	0.42	0.088	1.000
K601E	143	15	0.54	0.27	0.92	0.32	0.023	0.975
D594G	142	28	1.02	0.21	1.53	0.68	0.917	1.000
V600K	134	7	0.27	0.39	0.58	0.13	0.000	0.003
D594N	116	45	2.00	0.18	2.83	1.42	0.000	0.007
N581S	65	10	0.79	0.34	1.55	0.41	0.638	1.000
G466V	64	28	2.26	0.23	3.52	1.45	0.001	0.026
G466E	59	25	2.19	0.24	3.49	1.37	0.002	0.073
G469V	55	15	1.41	0.29	2.49	0.80	0.255	1.000
G469R	48	18	1.94	0.28	3.33	1.13	0.028	1.000
N581I	36	11	1.58	0.34	3.10	0.80	0.232	1.000
G469E	34	20	3.04	0.28	5.27	1.75	0.000	0.011
K601N	34	10	1.52	0.36	3.07	0.75	0.224	1.000
P403Lfs*8	31	15	2.50	0.31	4.63	1.35	0.008	0.326
S467L	29	14	2.49	0.33	4.72	1.32	0.007	0.293
G464V	27	9	1.72	0.38	3.66	0.81	0.172	1.000
L597R	27	5	0.96	0.49	2.48	0.37	1.000	1.000
BRAF-KIAA1549 fusion	55	1	0.09	1.01	0.68	0.01	0.112	1.000
T599dup	26	0	0.01	4.48	64.01	0.00	0.015	0.663
N486_P490del	26	0	0.01	4.48	64.01	0.00	0.015	0.663
V600R	25	0	0.01	4.48	66.59	0.00	0.025	1.000
G466A	24	6	1.29	0.46	3.16	0.53	0.618	1.000
E26D	23	11	2.47	0.37	5.06	1.20	0.018	0.768
G466R	23	7	1.57	0.43	3.66	0.67	0.318	1.000
A404Cfs*9	23	4	0.90	0.54	2.60	0.31	1.000	1.000
BRAF-SND1 fusion	22	0	0.01	4.48	75.74	0.00	0.038	1.000

© 2021 Zhao Y et al. *JAMA Network Open*.

	Count	Combined						
		Altered	OR	SE(ln(OR))	ci_upper	ci_lower	P_value	Q_value
G596R	20	5	1.29	0.50	3.44	0.48	0.587	1.000
V600_K601delinsE	20	1	0.26	1.02	1.92	0.03	0.235	1.000
L597Q	17	0	0.02	4.48	98.23	0.00	0.094	1.000
F595L	13	9	3.57	0.43	8.36	1.53	0.005	0.220
A762V	13	5	1.98	0.53	5.57	0.71	0.196	1.000
E501K	12	10	4.30	0.43	9.95	1.86	0.001	0.053
E586K	12	9	3.87	0.44	9.19	1.63	0.004	0.152
D594E	12	4	1.72	0.58	5.33	0.55	0.313	1.000
AGK-BRAF fusion	12	1	0.43	1.04	3.31	0.06	0.707	1.000
R389C	11	5	2.35	0.54	6.75	0.81	0.163	1.000
V600M	11	0	0.02	4.48	152.63	0.00	0.231	1.000
K483E	10	4	2.06	0.59	6.58	0.65	0.265	1.000
T241M	10	4	2.06	0.59	6.58	0.65	0.265	1.000
E695Q	10	2	1.03	0.77	4.71	0.23	1.000	1.000
L597S	10	1	0.52	1.05	4.03	0.07	1.000	1.000
G469S	10	0	0.03	4.48	168.15	0.00	0.383	1.000
R271H	9	5	2.87	0.56	8.56	0.96	0.063	N/A
D594Y	9	5	2.87	0.56	8.56	0.96	0.063	N/A
L485W	9	0	0.03	4.48	187.18	0.00	0.371	N/A
R354Q	8	5	3.23	0.57	9.86	1.05	0.046	N/A
L584F	8	4	2.58	0.61	8.57	0.78	0.116	N/A
D594H	8	2	1.29	0.79	6.08	0.27	0.670	N/A
BRAF-MKRN1 fusion	8	1	0.65	1.06	5.16	0.08	1.000	N/A
D594A	7	3	2.21	0.69	8.55	0.57	0.213	N/A
R671Q	7	2	1.47	0.80	7.10	0.31	0.646	N/A
S363F	6	4	3.44	0.65	12.19	0.97	0.064	N/A
G464R	6	2	1.72	0.82	8.52	0.35	0.624	N/A

© 2021 Zhao Y et al. *JAMA Network Open*.

	Count	Combined						
		**Altered**	**OR**	**SE(ln(OR))**	**ci_upper**	**ci_lower**	**P_value**	**Q_value**
L597V	5	5	5.16	0.63	17.83	1.49	0.014	N/A
P708S	5	3	3.10	0.73	12.96	0.74	0.127	N/A
R509*	5	3	3.10	0.73	12.96	0.74	0.127	N/A
D594V	5	3	3.10	0.73	12.96	0.74	0.127	N/A
N581T	5	2	2.06	0.84	10.64	0.40	0.318	N/A
F468S	5	1	1.03	1.10	8.83	0.12	1.000	N/A
G596C	5	1	1.03	1.10	8.83	0.12	1.000	N/A
BRAF-CDK5RAP2 fusion	5	0	0.05	4.49	341.91	0.00	1.000	N/A
AGAP3-BRAF fusion	5	0	0.05	4.49	341.91	0.00	1.000	N/A

Abbreviations:
OR: odds ratio
SE(ln(OR)): Standard Error of the Natural Log of the Odds Ratio
ci_upper: Upper limit of the 95% confidence interval
ci_low: Lower limit of the 95% confidence interval

© 2021 Zhao Y et al. *JAMA Network Open*.
